# Detection and Monitoring of Regulatory Immune Cells Following Their Adoptive Transfer in Organ Transplantation

**DOI:** 10.3389/fimmu.2020.614578

**Published:** 2020-12-14

**Authors:** Lillian M. Tran, Angus W. Thomson

**Affiliations:** ^1^ Thomas E. Starzl Transplantation Institute, Department of Surgery, University of Pittsburgh School of Medicine, Pittsburgh, PA, United States; ^2^ Department of Immunology, University of Pittsburgh School of Medicine, Pittsburgh, PA, United States

**Keywords:** cell therapy, adoptive transfer, cell tracking, regulatory T cell, mesenchymal stromal cell, regulatory myeloid cell, transplantation

## Abstract

Application of cell-based immunotherapy in organ transplantation to minimize the burden of immunosuppressive medication and promote allograft tolerance has expanded significantly over the past decade. Adoptively transferred regulatory immune cells prolong allograft survival and transplant tolerance in pre-clinical models. Many cell products are currently under investigation in early phase human clinical trials designed to assess feasibility and safety. Despite rapid advances in manufacturing practices, defining the appropriate protocol that will optimize *in vivo* conditions for tolerance induction remains a major challenge and depends heavily on understanding the fate, biodistribution, functional stability and longevity of the cell product after administration. This review focuses on *in vivo* detection and monitoring of various regulatory immune cell types administered for allograft tolerance induction in both pre-clinical animal models and early human clinical trials. We discuss the current status of various non-invasive methods for tracking regulatory cell products in the context of organ transplantation and implications for enhanced understanding of the therapeutic potential of cell-based therapy in the broad context of control of immune-mediated inflammatory disorders.

## Introduction

Cell-based therapy using naturally occurring or genetically modified immune cells, having now been successfully translated to the clinic for cancer treatment, is undergoing clinical development to promote tolerance and prolong graft survival after solid organ transplantation. Cell products under active investigation for clinical use in kidney or liver transplantation include donor-antigen alloreactive regulatory T cells (darTreg) and polyclonally expanded Tregs (pTreg), regulatory macrophages (Mreg), regulatory dendritic cells (DCreg), and mesenchymal stromal cells (MSCs). Findings from the recent ONE Study, the largest multi-center consortium to date assessing adoptive cell therapy in kidney transplant patients, have confirmed the safety of infusing various regulatory immune cells, paving the way for further development (1). The main challenge in clinical testing of regulatory cell therapy, however, is that the *in vivo* fate and localization of the cell product remains largely unknown which leads to major gaps in understanding of tolerance induction mechanisms and hinders cell therapy protocol design. Non-invasive, accurate, and durable techniques to monitor exogenous cell products after infusion in both pre-clinical and clinical human studies are critical in addressing 1) variability in clinical outcomes, 2) potential cell toxicity and adverse side effects of infusion, 3) anatomic localization and 4) duration and magnitude of desired tolerogenic activity (2).

Historically, cellular staining and subsequent flow cytometry has been a reliable approach to detecting adoptively transferred cells (3–5) however, more advanced approaches to longitudinal *in vivo* cell monitoring using whole-body imaging with novel reporter systems, initially developed for cancer immunotherapy, are increasingly being incorporated into both pre-clinical and clinical transplant studies. This review will discuss current techniques used to track and monitor the major regulatory immune cells under clinical investigation for tolerance induction (**Table 1**) and how understanding the in vivo fate of these cell products has helped advance cell-based therapy in organ transplantation.

**Table 1:** Methods used to track and monitor regulatory immune cells adoptively transferred for transplant indications.

Species	Cell type	Transplanted allograft	Detection method	Duration of tracking post-infusion (days)	Reference
**Rodent**					
	Tregs/autologous	Heart	Treg-specific mAb staining	98	(6)
	Tregs/donorAg-reactive	Skin	CSFE dye/GFP	60	(7)
	Tregs/autologous	Pancreatic islet	CSFE/PKH-26 dye	10	(8)
	Tregs/autologous and non-autologous	VCA	Luciferase gene-reporter system	294	(9)
	Tregs/autologous	Heart/Lung	IOPC-NH2/MRI	3	(10)
	Tregs/autologous	Skin	99mTcO4-/SPECT	1	(11)
	Tregs/CAR	Skin	Luciferase gene-reporter system	21	(12)
	Tregs/CAR	Pancreatic islet, skin	Luciferase gene-reporter system	21	(13)
	Mregs/donor-derived	Heart	Donor-discriminatory Mreg mAb staining	28	(14)
	DCregs/autologous	Heart	PKH-26 dye	5	(15)
	DCregs/donorAg-pulsed	Heart	PKH-67 dye	1	(16)
	DCregs/donor-derived	Heart	Donor-discriminatory DCreg mAb staining	7	(17)
	MSCs/autologous	Kidney	PKH-26 dye	1–2	(18)
	MSCs/donor-derived	Heart	PKH-26 dye	21	(19)
**NHP**					
	Tregs/autologous and non-autologous	–	CSFE/VPD450 dye	21	(20)
	Tregs/autologous	–	CSFE dye	40	(21)
	Tregs/autologous	–	CSFE/VPD450 dye	87	(22)
	Tregs/autologous	–	CSFE dye	100	(23)
**Human**					
	Tregs/autologous	Kidney	Deuterium labeling	180	(24)
	Mregs/donor-derived	Kidney	^111^In labeling/SPECT	1	(25)
	DCregs/donor-derived	Liver	Donor-specific MHC mAb staining	7	(26)

CAR, chimeric antigen receptor; NHP, non-human primates; Tregs, regulatory T cells; Mregs, regulatory macrophages; DCregs, regulatory dendritic cells; GFP, green fluorescence protein; VCA, vascularized composite allotransplantation; mAb, monoclonal antibody; donorAg, donor-antigen; CSFE, carboxyfluorescein succinimidyl ester; VPD450, violet proliferation dye 450; IOPC-NH2, superparamagnetic nano-sized iron-oxide particle; MRI, magnetic resonance imaging; 99mTcO4-, technetium-99m pertechnetate; SPECT, single-photon emission computed tomography; ^111^In, ^111^Indium tropolonate; MHC, major histocompatibility complex.

## Tracking/Monitoring of Polyclonal or Donor Ag Alloreactive Tregs IN Experimental Organ Transplantation

Naturally occurring Tregs are a rare, specialized subset of thymic-derived CD4^+^CD25^+^ T cells characterized by high expression of the transcription factor forkhead box P3 (Foxp3). In addition to these thymic Tregs (tTregs), naïve Foxp3^-^CD4^+^ T cells can differentiate in the periphery to become Foxp3^+^ cells, that are known as induced Tregs (iTregs) or peripheral Tregs. Distinctions between tTregs and iTregs have been reviewed recently (27). T cell receptors (TCRs) that recognize antigens to which an organism is chronically exposed promote the generation of iTregs (28, 29).

Tregs have been implicated extensively in tolerance induction and maintenance pathways. Their potential to regulate allograft rejection after transplantation is the most extensively evaluated of the regulatory cell types under current investigation. Given their paucity in the peripheral circulation in the healthy steady state, one salient issue in using Tregs to promote tolerance induction is whether these cells can persist or self-expand in host peripheral blood and tissue to exert a sustained therapeutic effect after administration. Localization and physical distribution of Tregs within allograft tissue, in particular, have been associated with enhanced immunomodulatory function *in vivo*, implicating the importance of cell homing in adoptive Treg cell therapy for transplant tolerance (30–32). As such, methodologies to track the fate of infused Tregs are critical and have been incorporated into both pre-clinical and early clinical studies (**Table 2**).

**Table 2:** Observations of adoptively transferred Treg survival and migration in various species.

Species	Cell origin	Transplant allograft	Sites of cell trafficking	Duration of *in vivo* detection post-infusion	Comments	Reference
**Rodent**						
	Autologous	Heart	Peripheral blood, spleen, mesenteric LN, allograft	98 days	Tregs detected in blood at day 7 and 98, all others at day 98	(6)
	Autologous, donorAg-pulsed	Skin	Spleen, draining and mesenteric LN, allograft	21 days (spleen) 60 days (LNs, allograft)		(7)
	Autologous	Pancreatic islet	Spleen, draining and non-draining LN, allograft	4 days	Tregs migrate first to allograft then to LNs	(8)
	Autologous and non-autologous	VCA	Axillary and inguinal LNs, allograft	4–294 days (non-autologous) 4–14 days (autologous)	Tregs migrate first to LNs (day 4) then to allograft (day 6); Tregs failed to persist after 2 weeks in syngeneic recipients	(9)
	CAR and non-autologous	Skin	Allograft, draining LN	2–21 days (CAR) 2–7 days (non-autologous)	Polyclonal Tregs homed to both HLA- A2-expressing allograft and non-A2 skin while CAR Tregs homed to A2-expressing skin allograft only	(12)
	CAR and non-autologous	Pancreatic islet, skin	Islet and skin allograft, draining LN, spleen	1–21 days (CAR)	FITC-H-2D^d^ -mAbCAR Tregs show enhanced localization to the islet allograft.	(13)
	Autologous	Heart/Lung	Allograft	24–48 h	Labeled Tregs detected in both heart and lung allograft on MRI	(10)
	Autologous	Skin	Spleen, liver, intestines, heart, tail, thymus, muscle	24 h	Only study to demonstrate uptake of labeled Tregs in non-lymphoid tissues	(11)
**NHP**						
	Autologous and non-autologous	–	Peripheral blood	21 days (autologous) 6 days (non-autologous)	In non-transplanted model, auto Treg survival higher than MHC-mismatched Treg	(20)
	Autologous	–	Peripheral blood, bone marrow (BM), LNs	16 days (peripheral blood) 37 days (peripheral blood, + rapamycin) 13 days (BM) 6 days (LN)	Rapamycin therapy enhanced *in vivo* persistence of infused Tregs in blood	(21)
	Autologous	–	Peripheral blood, spleen, inguinal LN, mesenteric LN	71 days (peripheral blood) 50 days (lymphoid tissue)		(22)
**Human**						
	Autologous	Kidney	Peripheral blood	90 days	Infused Tregs peaked at 2–8% of total Tregs in peripheral blood dropping below detection by 3 months	(24)

BM, bone marrow; CAR, chimeric antigen receptor; FITC, fluorescein isothiocyanate; LN, lymph node; mAb, monoclonal antibody; MRI, magnetic resonance imaging; NHP, non-human primates; Tregs, regulatory T cells; VCA, vascularized composite allotransplantation; MHC, major histocompatibility complex.

Early studies investigating *ex vivo-*expanded polyclonal Tregs adoptively transferred to skin- or pancreatic islet-engrafted mice relied on direct labeling of the *ex vivo*-expanded CD4^+^CD25^+^Foxp3^+^ cells with intracellular carboxyfluorescein succinimidyl ester (CFSE) dye or on Treg generated from green fluorescent protein (GFP) transgenic donors to study tissue homing and survival of these cells after intravenous administration (6, 7). Flow cytometric analysis detected labeled autologous (6) or darTregs (7) in peripheral blood, spleen, draining lymph nodes (LN) and allograft tissue, up to 60 to 98 days post-infusion demonstrating the persistence and trafficking of adoptively transferred autologous Tregs to secondary lymphoid organs. These studies also determined the cell surface molecules integral to Treg migration. E and P selectin ligands were found to be important in Treg homing to the graft, while chemokine receptors CCR7, CCR2, and CCR5 were required for their migration to secondary lymphoid tissue (8).

More recent studies in transplanted rodents have shifted towards non-invasive whole-body *in vivo* cell tracking and imaging of adoptively transferred Tregs. Tregs isolated and expanded *ex vivo* from luciferase transgenic rats were adoptively transferred into major histocompatibility complex (MHC)-mismatched vascularized composite allotransplant rat recipients and visually tracked using bioluminescence imaging (BLI) longitudinally. In contrast to the limited detection of labeled Tregs in cross-sectional samples of earlier studies, real-time *in vivo* imaging allowed Cheng et. al to identify migratory patterns of infused Tregs first to draining LNs and then to grafted tissue over a prolonged period of 42 weeks (9). A novel method using superparamagnetic nano-sized iron-oxide particle, IOPC-NH2, to label transferred T cells and magnetic resonance imaging (MRI) was developed by Liu et. al and successfully demonstrated localized infiltration of IOPC-NH2-labeled autologous T cells into allograft tissue within 24 h in a rat heart-lung transplant model (10). Radiolabeling of *ex vivo*-expanded Tregs with technetium-99m pertechnetate (99mTcO4^-^) was performed both directly and indirectly via retroviral transduction with a construct expressing the hNIS glycoprotein ion channel gene (11). These studies localized adoptively transferred labeled Tregs in spleen, liver, lungs, and the allograft after administration and skin transplantation in mice with the approach allowing longitudinal detection of transferred Tregs *in vivo* over time.

Tregs have been well-characterized in nonhuman primates (NHP) (20, 21, 33–35).The *in vivo* persistence and homing of adoptively transferred pTregs to secondary lymphoid organs demonstrated in rodent models have been corroborated by several NHP studies evaluating the survival, migration, and function of exogenous Tregs after administration. *In vivo* detection of *ex vivo*-expanded autologous or allogeneic Tregs infused systemically into non-transplanted cynomolgus or rhesus macaques was accomplished through direct CSFE or violet proliferation dye 450 (VPD450)-labeling and subsequent flow cytometric analysis of the labeled cells in peripheral blood, mesenteric and inguinal LNs, and spleen at various timepoints post-infusion (20, 21). Pharmacokinetic analysis of CSFE-labeled autologous Tregs detected an initial rapid phase of elimination from the peripheral blood between day 0 and day 3 post-infusion after which these transferred cells persisted at low levels in the blood up to 3 weeks (21). Persistence of these cells in secondary lymphoid organs was not as durable. Labeled autologous Tregs were detected in inguinal and mesenteric LNs harvested at days 1 to 2 post-infusion, but lost to detection by day 6 (21). Administration of concurrent immunosuppression (IS) therapy substantially increased survival of transferred autologous Tregs in peripheral blood and LNs. Labeled autologous Tregs persisted longer in peripheral blood and LNs in monkeys given rapamycin alone or with concurrent IL-2 and were detected in these compartments in greater numbers when compared to non-immunosuppressed conditions 50 to 84 days post-infusion (22, 23). These studies highlight the wide variability in survival of infused Tregs under numerous different conditions, including the presence and type of IS, as well as cell production techniques, particularly cryopreservation.

Studies in splenectomized, kidney-transplanted NHP treated early post-transplant with cyclophosphamide and then infused with *ex vivo*-expanded autologous Tregs support their efficacy in prolonging allograft survival and function. In addition, multiple Treg infusions in NHP pretreated with anti-thymocyte globulin (ATG) and post-operative rapamycin prolonged renal allograft survival (36). In contrast, Ezzelarab et. al failed to demonstrate enhanced heart allograft survival after adoptive transfer of autologous pTreg to ATG-treated heart allograft recipients, possibly reflecting, in part, reduced survival capacity of the pTreg product *in vivo* (37). Overall, *in vivo* detection of the transferred regulatory cells in the majority of these studies was limited, as they focused primarily on allograft survival outcomes.

## Tracking/Monitoring of CAR Tregs in Experimental Organ Transplantation

While the majority of pre-clinical studies investigating the efficacy of Treg cell therapy have focused on polyclonal autologous and non-autologous Tregs, several groups have evaluated the potential of using chimeric antigen receptor (CAR) modified Tregs as a more potent and targeted cellular method of tolerance induction after transplantation. Investigators have demonstrated that adoptive transfer of genetically engineered donor HLA-specific CAR Tregs successfully prevents the rejection of transplanted allogeneic cells and graft tissue in humanized mouse models (12, 13, 38, 39). *In vivo* BLI utilizing the luciferase-GFP reporter system showed rapid and specific trafficking of adoptively transferred HLA-A2-specific CAR Tregs (12) or mAb-directed CAR Tregs targeted to H-2D^d^ (13) to transplanted skin or pancreatic islet allografts respectively, persisting up to 21 days after transfer. Additionally, both studies demonstrated that, compared to their polyclonal counterparts, CAR Tregs achieved a more targeted localization and longer persistence in allograft tissue.

## Tracking/Monitoring of Tregs in Human Organ Transplantation

In humans, early phase clinical testing of adoptively transferred autologous Tregs in transplant patients is well underway. Deuterium-labeled autologous pTregs were infused and tracked in the peripheral blood of 3 kidney transplant recipients on maintenance IS regimen of tacrolimus, mycophenolate mofetil ± prednisone with subclinical inflammation on 6-month surveillance biopsy (24). CD4^+^CD127^lo^CD25^+^ Tregs were purified via FACS from peripheral blood and single cell suspensions from kidney biopsies. DNA was then extracted from all purified cells and subjected to gas chromatography and mass spectrometry (GC-MS) analysis to measure deuterium enrichment in circulating Tregs. Infused Tregs peaked within 7 days of infusion and were detected by deuterium signals at 30 days. Deuterium-labeled cells fell to the limit of detection within 3 months of infusion (24). In this study, infused Tregs demonstrated patterns of persistence and stability comparable to those observed in prior corresponding immunosuppressed NHP models and non-immunosuppressed type 1 diabetes mellitus patients receiving autologous pTreg therapy (40). Anatomic biodistribution of clinical grade Tregs after therapeutic infusion was ascertained in a non-transplant autoimmune hepatitis (AIH) clinical pilot study by radiolabeling of good manufacturing practice (GMP)-grade Tregs with ^111^Indium tropolonate (^111^In) (41). Serial gamma camera and SPECT-CT imaging taken at serial timepoints after infusion tracked the presence of transferred indium-labeled Tregs. 22% to 44% of infused Tregs migrated to the liver, spleen and bone marrow of 4 AIH patients for up to 72 h without any off-target organ localization (41). This provides an additional effective cell tracking method that can be implemented in current and future transplant Treg therapy human clinical studies to assess spatial distribution of infused cell therapy non-invasively in real-time.

## Tracking/Monitoring of Regulatory Myeloid Cells in Experimental Organ Transplantation

The myeloid cell lineage includes multiple regulatory immune cell subsets under active investigation to induce and maintain transplant tolerance in solid organ transplantation, including DCregs, Mregs, and myeloid-derived suppressor cells (MDSCs) (42) (**Table 3**).

**Table 3:** Observations of adoptively transferred regulatory myeloid cell survival and migration in various species.

Species	Cell type	Cell origin	Transplanted allograft	Sites of cell trafficking	Duration of *in vivo* detection post-infusion	Comments	Reference
**Rodent**							
	Mregs	Donor-derived	Heart	Peripheral blood, spleen, LN, BM, liver, lung	14 days	24-h post-infusion, infused Mregs detected most in lung/liver, but dissipate thereafter	(14)
	DCregs	Autologous	Heart	Spleen	5 days		(15)
	DCregs	donorAg-pulsed	Heart	Spleen	24 h		(16)
	DCregs	Donor-derived	Heart	Spleen	24 h		(17)
**Human**							
	Mregs	Donor-derived	Kidney	Lung, liver, spleen, BM	30 h	Majority of labeled infused Mregs detected in lungs, then dissipate to liver and spleen after 2.5-h post-infusion	(25)
	DCregs	Donor-derived	Liver	Donor-specific MHC mAb staining	1 h (intact) 7 days (donorAg)	Intact infused DCreg were not detected after 1-h post-infusion, however donor-specific Ag detected on recipient DC up to 7 days	(26)

BM, bone marrow; DCregs, regulatory dendritic cells; Mregs, regulatory macrophages; mAb, monoclonal antibody.

MDSCs comprise a heterogeneous population of immature myeloid progenitor cells that have been associated with modulation of T cell differentiation. There is evidence from pre-clinical rodent models that MDSCs may play a role in promotion of transplant tolerance by inducing Treg and inhibiting alloreactive T cell proliferation in an inducible nitric oxide synthase (iNOS)-dependent manner (43, 44) however, adoptive transfer of ex vivo-generated MSDCs has not been found in pre-clinical animal studies to improve allograft survival (45) and, as such, has not reached clinical testing in humans to date (46). Tracking of infused MDSCs has thus far been restricted to mouse cancer models with one study using a ^64^Cu-labeled CD11b-specific mAb and PET scanning (47).

Mregs, characterized by a CD14^-^CD63^+^HLA-DR^+^ phenotype and IL-10 production, have been demonstrated to suppress T cell proliferation *in vitro* (48). In a heterotopic heart transplant mouse model, administration of donor-specific Mregs significantly prolonged allograft survival in an iNOS-dependent manner (14). Mregs were tracked *in vivo* using donor-discriminatory Mreg staining and flow cytometry analysis of cells from recipient blood, spleen, liver, LN, BM, and lung suspensions at serial timepoints post-infusion. 24 h after administration, Mregs were readily detected in the blood, spleen, liver, and lung but not in LN or BM. Persistence of infused Mregs decreased in all tissue compartments thereafter up to 2 weeks, after which Mregs were no longer detectable (14). Notably, cross-dressing of recipient antigen-presenting cells (APCs) with donor-specific Ag was not observed in this study.

DCregs are another myeloid-derived immune cell subset whose tolerogenic properties have been well-characterized (49) and have thus, garnered significant attention for clinical testing and use in transplant tolerance induction therapy (50). Extensive pre-clinical testing in organ- and skin transplanted mouse models, has demonstrated that the adoptive transfer of *ex vivo-* generated autologous or donor-derived DCreg prolongs allograft survival and promotes donor Ag- specific tolerance. These effects have been achieved either in the absence of, or in combination with, short-term IS (51–57). Two reports have suggested that donor-derived DCreg can prime the alloimmune response (58, 59). In a heterotopic cardiac transplantation rat model, infused autologous DCregs were labeled with PKH-26 red fluorescent cell linker which allowed their detection in spleens of recipient rats using immunofluorescence imaging of histological sections 5 days after administration (15). The use of additional fluorochromes allowed elucidation of interferon-gamma production induction as a potential mechanism of immunoregulation. The lipophilic membrane dye PKH was also used to label rapamycin-treated autologous DCregs pulsed with alloAg that were also administered to heart transplanted mice (16). DCreg homing to spleen was unaffected by rapamycin treatment, but conferred the capacity to suppress alloAg-specific T cell proliferation. Donor discriminatory MHC staining and flow cytometry analysis have also been utilized to detect *in vivo* survival of infused donor-derived DCregs in heart-transplanted mice, which has been shown to be short-lived likely due to killing/removal by host natural killer (NK) cells (60). Thus, the therapeutic effect of pre-transplant infusion of donor-derived DCreg does not appear to depend on the in vivo persistence of intact donor DCreg which offers a potential advantage over other cell therapy approaches for which immunosuppressive ability that may depend on in vivo persistence of the transferred regulatory cells.

## Tracking/Monitoring of Regulatory Myeloid Cells in Human Organ Transplantation

In a human study published in 2011, two kidney transplant recipients were infused with donor-derived Mreg pre-operatively and shown to successfully wean to low-dose tacrolimus monotherapy within 24 weeks of transplantation, with no evidence of adverse effect or rejection (25). A small proportion (12%) of adoptively transferred Mregs were radiolabeled using ^111^In prior to infusion allowing for *in vivo* Mreg tracking in real-time using SPECT-CT scanning. Scintigrams reconstructed from SPECT imaging demonstrated initial trapping of labeled Mregs in the pulmonary vasculature, but after 2.5 h re-distributed to the peripheral blood, liver, and spleen. 24 h after infusion, Mregs were no longer detectable in the lungs or peripheral blood and were seen to accumulate in lymphoid and non-lymphoid organs (25). Pre-transplant administration of Mreg therapy in two enrolled kidney transplant recipients was most recently assessed for safety and feasibility as part of the multi-center ONE study, however efficacy and *in vivo* cell tracking/distribution were not evaluated (1, 61).

Donor discriminatory HLA staining is being used to track donor-derived DCregs infused 7 days before transplant into prospective living donor liver transplant recipients. Detection of the donor DCreg and their products is enhanced by image-based flow cytometry methods that can directly visualize the expression of MHC Ags and other gene products of donor or recipient origin by APCs in the circulation and host lymph nodes (26).

## Tracking/Monitoring of MSCs in Experimental Organ Transplantation

MSCs are naturally occurring, bone marrow-derived precursor cells, unique in their activation and migration to inflammatory sites, including allograft tissue, where they can exert their immunoregulatory effects, including upregulation of Treg differentiation in the inflammatory microenvironment (48). Administration of *ex vivo*-expanded MSCs has now consistently proven to be effective in prolonging allograft survival in murine models of solid organ transplantation (62). For *in vivo* tracking, cell labeling with PKH -26 red fluorescence cell linker has been used in murine models infused with autologous or donor-derived MSCs 7 days before kidney or semi-allogeneic heart transplantation (18, 19). In kidney allografted mice, adoptively transferred autologous MSCs infused 1 day prior to kidney transplantation preferentially migrated to the spleen, correlated with better graft survival, whereas post-transplant administration of MSCs was associated with infiltration of the allograft and subsequent C3 complement deposition without any therapeutic effect on allograft function (18). In cardiac allografted mice, PKH-26+ donor-derived MSCs infused prior to transplantation localized to liver, lung, primary and secondary lymphoid organs after infusion with none detected in peripheral blood. Survival in lymphoid tissue and lung was short-lived as PKH-26+ MSCs were not detected in these compartments at day 7 and 21 timepoints, while transferred MSCs were still detected in liver at day 7 post-infusion.

## Tracking/Monitoring of MSCs in Human Organ Transplantation

Multiple human studies investigating the safety, feasibility, and efficacy of adoptively transferred MSCs in solid organ transplantation are currently ongoing (63–65). One large randomized, controlled trial using MSC-based induction therapy in living donor kidney transplantation has already demonstrated reduced incidences of acute rejection, lower rates of infection, and improved 1-year graft function (63). Cell tracking/localization experiments in published human studies are lacking, however the importance of tissue localization following MSC administration is bound to prompt current or future human studies to incorporate non-invasive in vivo detection methods of this infused regulatory cell product.

## Conclusions

Cell-based therapies are increasingly being considered and investigated for minimization of IS and induction/maintenance of tolerance in solid organ transplantation. As such, gaps in understanding of the *in vivo* fate of adoptively transferred regulatory immune cells after administration need to be filled in order to advance translation of these treatments to the clinic. Current direct cell labeling and flow cytometric analyses of target cells using intracellular dyes or surface marker tags have been efficacious in determining persistence of transferred cells in pre-clinical animal models; however, they lack anatomic information and are cumbersome to apply routinely to human studies due to the need for frequent blood draws and/or tissue biopsies. Cell radiolabeling in conjugation with imaging modalities such as SPECT or MRI has proven to be a more effective strategy of longitudinal *in vivo* cell monitoring in humans given its non-invasive approach, but commonly used radionuclides are often severely limited by their short half-lives. Advanced multi-modal approaches utilizing a dual reporter gene/radiolabeling system and whole-body imaging would provide the highest resolution and sensitivity of monitoring infused cell therapy in the most comprehensive and non-invasive way.

As current early phase human studies investigating various regulatory immune cell products for transplant tolerance advance to higher stages of clinical testing, incorporating some method of *in vivo* monitoring of the infused regulatory cell products without detriment to their function/survival will become imperative to ensure patient safety and maximize therapeutic potential.

## Author Contributions

Both authors contributed to the content design, literature searches, writing of the manuscript, and manuscript review. All authors contributed to the article and approved the submitted version.

## Funding

Work described in this article was supported in part by National Institutes of Health (NIH) grants R01 AI118777, U01 AI 136779, and U19 AI131453 to AT, and by an NIH institutional research training fellowship to LT (T32 AI074490). Additionally, the University of Pittsburgh holds a Physician-Scientist Institutional Award from the Burroughs Wellcome Fund, which supports LMT.

## Conflict of Interest

The authors declare that the research was conducted in the absence of any commercial or financial relationships that could be construed as a potential conflict of interest.
